# OptCouple: Joint simulation of gene knockouts, insertions and medium modifications for prediction of growth-coupled strain designs

**DOI:** 10.1016/j.mec.2019.e00087

**Published:** 2019-03-16

**Authors:** Kristian Jensen, Valentijn Broeken, Anne Sofie Lærke Hansen, Nikolaus Sonnenschein, Markus J. Herrgård

**Affiliations:** a The Novo Nordisk Foundation Center for Biosustainability, Technical University of Denmark, Building 220, Kemitorvet, 2800 Kgs. Lyngby, Denmark

## Abstract

Biological production of chemicals is an attractive alternative to petrochemical-based production, due to advantages in environmental impact and the spectrum of feasible targets. However, engineering microbial strains to overproduce a compound of interest can be a long, costly and painstaking process. If production can be coupled to cell growth it is possible to use adaptive laboratory evolution to increase the production rate. Strategies for coupling production to growth, however, are often not trivial to find. Here we present OptCouple, a constraint-based modeling algorithm to simultaneously identify combinations of gene knockouts, insertions and medium supplements that lead to growth-coupled production of a target compound. We validated the algorithm by showing that it can find novel strategies that are growth-coupled in silico for a compound that has not been coupled to growth previously, as well as reproduce known growth-coupled strain designs for two different target compounds. Furthermore, we used OptCouple to construct an alternative design with potential for higher production. We provide an efficient and easy-to-use implementation of the OptCouple algorithm in the cameo Python package for computational strain design.

## Highlights

• A mixed integer linear program is used to couple bio-production to cell growth.
• Designs consist of combinations of knockouts, insertions and medium supplements.
• Published and experimentally validated growth-coupled designs can be predicted.
• Strain designs for coupling three different chemicals to cell growth are predicted.

## 1 Introduction

The use of microorganisms as cell factories offers the possibility of producing a wide range of chemicals from renewable sources, as well as manufacturing natural compounds too complicated for chemical synthesis in large amounts (Becker and Wittmann, 2015). However, successfully engineering microorganisms to produce a target compound most often requires trial-and-error experimentation with different possible pathways, and even when production is achieved, many iterations of subsequent optimization are usually necessary to increase production rate and yield to satisfy industrial needs (Lee and Kim, 2015).

One strategy for optimizing chemical production in microbial strains is to utilize the power of natural selection in adaptive laboratory evolution (ALE) experiments (Portnoy et al., 2011; Shepelin et al., 2018). This allows the identification of mutant strains with enhanced viability under the evolution conditions. The inherent selection for cells that are able to grow faster than the rest of the population makes it easy to optimize for characteristics such as product tolerance or substrate utilization, while directly improving production characteristics such as production rate, titer and yield is more difficult (Hansen et al., 2017; Shepelin et al., 2018). Indeed, with the advent of more and more methods, models, and databases for automated running and analysis of ALE experiments, such as eVOLVER (Wong et al., 2018), ALEsim (LaCroix et al., 2017), and ALEdb (Phaneuf et al., 2018), the need for new selective pressures by clever strain and experimental design becomes the primary challenge for evolutionary strain engineering.

Using evolution to improve biochemical production rates can be achieved by coupling production to growth, i.e. ensuring that production is a necessary by-product of cell growth, such that adaptations that increase the growth rate of the cells will also increase production. For a review of examples of successful growth-coupling for biochemical production, see e.g. Shepelin et al. (2018). A recent successful example is the growth-coupling of itaconic acid production in *Escherichia coli* by four gene deletions, a downregulation, and glutamate supplementation that ensure formation of itaconic acid to prevent accumulation of PEP inside the cell (Harder et al., 2016). The design was aided by the computation of minimal cut sets (MCS), which are sets of gene knockouts that will prevent all undesirable flux distributions while maintaining the ability to produce the target compound (Klamt and Gilles, 2004; von Kamp and Klamt, 2014).

Since growth-coupling strategies are not always obvious from looking at a metabolic map of the microorganism, it is beneficial to use genome-scale metabolic models together with computational methods like the MCS framework, to quickly search the design space for strain modifications that can potentially make production growth-coupled. One of the first computational methods for predicting strategies for improving bio-production was OptKnock (Burgard et al., 2003). OptKnock uses a mixed integer linear programming (MILP) formulation to predict gene knockouts that allow higher production under growth-optimal conditions. While the predictions made by OptKnock will allow for increased production, they will not necessarily make production growth-coupled, as alternative pathways can be used instead. The algorithm RobustKnock (Tepper and Shlomi, 2009) seeks to solve this problem by predicting knock-out combinations that maximize the minimal production under optimal growth. The more recent algorithm gcOpt (Alter et al., 2018) is similar to RobustKnock, but requires a fixed growth rate to be set, allowing the formulation to be simplified. In addition to finding gene knockouts, there are also algorithms, e.g. the RobOKoD algorithm (Stanford et al., 2015), that attempt to increase production rates by predicting native genes to under- and overexpress. However, growth-coupling a production pathway alleviates the need for such expression level perturbations, since these can be optimized subsequently by means of ALE (Shepelin et al., 2018).

It has been shown that almost all metabolites in *E. coli* can be growth-coupled through knockouts, but in many cases this would require deletion of an infeasible number of genes (von Kamp and Klamt, 2017). Growth coupling may be easier to achieve by inserting heterologous genes that alter host metabolism in addition to knocking out native genes. The algorithm OptStrain (Pharkya et al., 2004) predicts both knockouts and insertions for increasing production, but does so in a two-step process. First, heterologous reactions that enable or improve the production capabilities are identified from a database of known reactions. This can be a novel production pathway or stoichiometrically favourable alternate reactions. Subsequently, knockouts that increase the possible production yield at maximal growth are identified using the OptKnock algorithm. With a two-step procedure like OptStrain, it is only possible to find heterologous genes and knockouts that improve production independently of each other. To solve this problem the algorithm SimOptStrain (Kim et al., 2011) does simultaneous prediction of gene insertions and knockouts. This enables the identification of heterologous gene insertions that have beneficial effects, only in the presence of specific knockouts. An example of a design where heterologous genes and knockouts are combined is the growth-coupling of product methylation in a cysteine auxotrophic *E. coli* strain described by Luo and Hansen (2018). Insertion of *CYS3* and *CYS4* from *Saccharomyces cerevisiae* enable cysteine synthesis from supplemented methionine through a pathway that requires flux through S-adenosylmethionine (SAM)-dependent methyltransferase reactions. As seen in this design as well as the previously mentioned itaconic acid production design, growth-coupling strategies can result in auxotrophies, such that the growth medium must be supplemented with additional nutrients, i.e. methionine and glutamate, respectively. Although auxotrophies are generally undesirable in production processes as the addition of a supplement can incur a significant extra cost, auxotrophic growth-coupled strains can still be very useful in the strain development phase, particularly in combination with ALE (Shepelin et al., 2018). The recent algorithm SelFi (Hassanpour et al., 2017) attempts to couple growth to the flux catalysed by a target enzyme by constructing a carbon supply pathway including the target reaction and disabling alternative carbon supply pathways. This is done using a combination of knockouts and heterologous gene insertions as well as medium supplements. However, similar to OptStrain this is done in a two-step process, potentially excluding some designs. Furthermore, since growth coupling is achieved by constructing a new carbon supply pathway, the scope of target reactions is limited to reactions that can feasibly be incorporated into such a pathway.

Here we introduce OptCouple, an algorithm that simultaneously finds gene knockouts, insertions and modifications to the growth medium that result in coupling the production of a target chemical to growth in microorganisms. We have validated OptCouple by showing that it can predict known successful growth-coupling designs for the common production host *E. coli* and have used it to predict novel growth-coupling strategies.

## 2 Materials and methods

All computations were carried out in Python 3.6.4. A list of installed packages and an implementation of the entire prediction workflow, and scripts for the described analyses can be found in the supplementary material. Simulations were done using the iJO1366 genome-scale reconstruction of *E. coli* (Orth et al., 2011) as well as the reduced EColiCore2 model (Hädicke and Klamt, 2017). Simulations were performed with a maximum glucose uptake rate of 10 mmol/gDW/h and a maximum oxygen uptake of 1000 mmol/gDW/h.

### 2.1 MILP-based optimization of growth-coupling potential

The following section will go through the mathematical optimization problem forming the core of OptCouple. For the full mathematical formulation, see supplementary materials.

Growth-coupling potential can be defined as the increase in maximal growth rate obtained when allowing flux through the target reaction, i.e. the reaction producing the chemical of interest.

The symbol *M* is used to denote a full metabolic model with metabolites $m_{i}\forall \;i\in N$ and reactions $r_{j}\;\forall \;j\in R$, the target reaction, $r_{target}$, with the biomass reaction, $r_{biomass}$, as the objective function, while the symbol M^*^ is used to denote the metabolic model without the target reaction.

If we use *f* to denote objective function of a problem, the growth-coupling potential, *U*, can be mathematically described as:

(1) $$\begin{array}{c} U=\hat{f}\left(M\right)-\hat{f}\left(M^{*}\right) \end{array}$$

where $\hat{f}$ is used to denote the optimal objective value of a problem.

Every linear optimization problem can be converted into a dual problem (Ignizio and Cavalier, 1994), which will be denoted by a *D*-subscript, i.e. *M*_*D*_. One property of duality in linear optimization is that the dual problem will have the same optimal objective value as the primal, however if *M* is a maximization problem, *M*_*D*_ will be a minimization problem, and vice versa.

Each potential perturbation, i.e. gene knock-out, knock-in, as well as addition of a growth medium supplement, can be represented by a binary variable, $y_{j}\in Y\;\;\forall \;\;j\in R$, controlling the flux of the reaction associated with the given perturbation, i.e. native reactions, heterologous reactions and exchange reactions, for knockouts, knock-ins and medium supplements, respectively. Additional coupling constraints are added to ensure that a given reaction can only carry flux when its corresponding perturbation variable, $y_{j}$, has a value of 1 (see supplementary material).

The goal is to formulate an optimization problem that optimizes *U*, by finding an optimal combination of values for the control variables, *Y* and reaction fluxes, *v*:

(2) $$\begin{array}{c} Maximize_{Y,v}\;\hat{f}\left(M\right)-\hat{f}\left(M^{*}\right) \end{array}$$

This can be formulated as a bi-level optimization problem:(3)
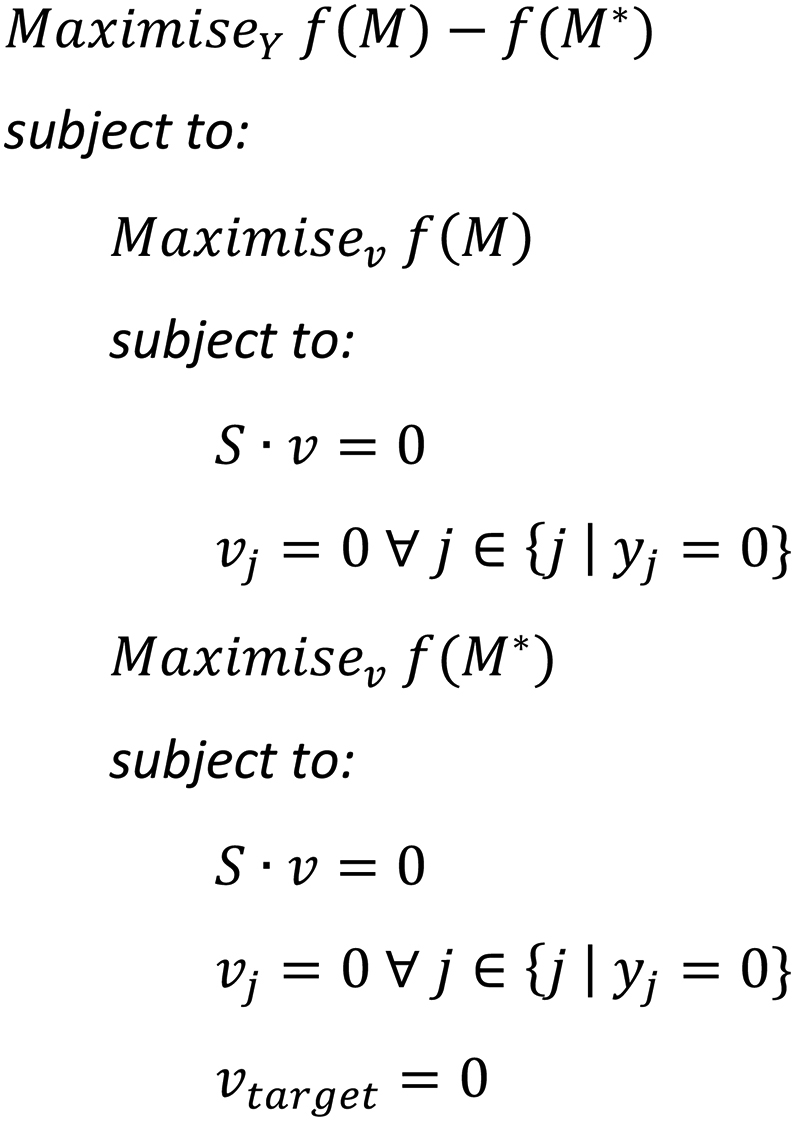


The bi-level formulation can be interpreted as finding the combination of control values that allows the highest growth-coupling potential, subject to the constraints that the fluxes (*v*) of *M* and M^*^ must be optimal for growth (under the given control variable values). The two inner optimization problems are fully specified in equations (S1) and (S2) in the supplementary material.

The bi-level formulation can be converted into a single optimization problem by replacing M^*^ with its dual form, M^*^_D_:

(4) $$\begin{array}{c} Maximize_{Y,v}\;f\left(M\right)-\;f\left(M_{D}^{*}\right) \end{array}$$

Since *M* is a maximization problem and M^*^_D_ is a minimization problem, maximizing this expression automatically ensures that $f\left(M\right)=\hat{f}\left(M\right)$ and $f\left(M_{D}^{*}\right)=\hat{f}\left(M_{D}^{*}\right)$, and since the optimal objective value of a dual problem is the same as the optimal objective value of its primal, the expression $\hat{f}\left(M\right)-\hat{f}\left(M_{D}^{*}\right)$ still corresponds to the growth-coupling potential. A full mathematical formulation of equation (4) can be found in equation (S4) in the supplementary material.

To maintain computational feasibility of the problem, maximum numbers of knock-outs, insertions and media modifications, respectively, can be set as constraints on the binary variables.

OptCouple is implemented in the *cameo* Python package (Cardoso et al., 2018) for computational strain design (https://github.com/biosustain/cameo), and an implementation can also be found in the supplementary material.

### 2.2 Selecting allowed gene insertions and medium supplements

The set of allowed heterologous gene insertions was obtained from metanetx (Moretti et al., 2016), through the universal model interface of the Python package *cameo* (Cardoso et al., 2018). Only reactions with a cross-reference to the BiGG database were used. To avoid drastically increasing running times due to the large pool of heterologous reactions, the list of allowed insertions was reduced according to the number of allowed simultaneous insertions. If a single insertion was allowed, only reactions with metabolites native to the host were allowed. For higher numbers of allowed insertions, the heterologous reaction network was pruned such that only reactions whose metabolites could be reached with the allowed number of inserted reactions were included. The list of allowed medium modifications is specified manually. For all predictions described in this work the list comprised fructose, lactate, acetate, and all 20 standard proteinogenic L-amino acids.

### 2.3 Running MILP optimizations

The MILP problems were optimized using the Gurobi solver (ver 7.5.2) through the *optlang* interface (Jensen et al., 2017). The computations were run on nodes of an HPC cluster equipped with Intel Xeon 2660v3 processors and 128 GB memory. The problems were solved to optimality, and subsequently reoptimized using Gurobi's solution pool feature to collect additional optimal and sub-optimal integer solutions. The second optimization was run with a time-limit approximately ten times the running time of the first optimization, up to a maximum of 30 h. For problems that could not be solved to optimality within 30 h, only as many suboptimal solutions as possible were collected from the second run. Each problem was optimized multiple times and the identified solutions from each run were all pooled together to increase the number of obtained solutions. Since the identification of integer solutions is not deterministic, and since multiple solutions from the same run tend to be similar, this allowed a more diverse sampling of the solution space.

### 2.4 Reducing solution redundancy

With other MILP-based algorithms like OptKnock (Burgard et al., 2003), a common practice is to gradually increase the number of allowed knockouts, to avoid getting solutions with unnecessary knockouts. With three different upper limits on modifications (for knockouts, gene insertions and medium supplements, respectively), such a strategy is significantly more time-consuming. Instead, a postprocessing workflow was used to identify the predicted modifications in each solution that do not contribute to growth-coupling. Each solution was simulated, and each modification was removed one at a time. If a modification could be excluded without eliminating growth-coupling, it was removed from the solution. Solutions that could be reduced to the same set of modifications were merged into a single solution. The remaining solutions were summarized by production and growth rates, as well as a production envelope plot.

## 3 Calculation

**Fig. 1:**
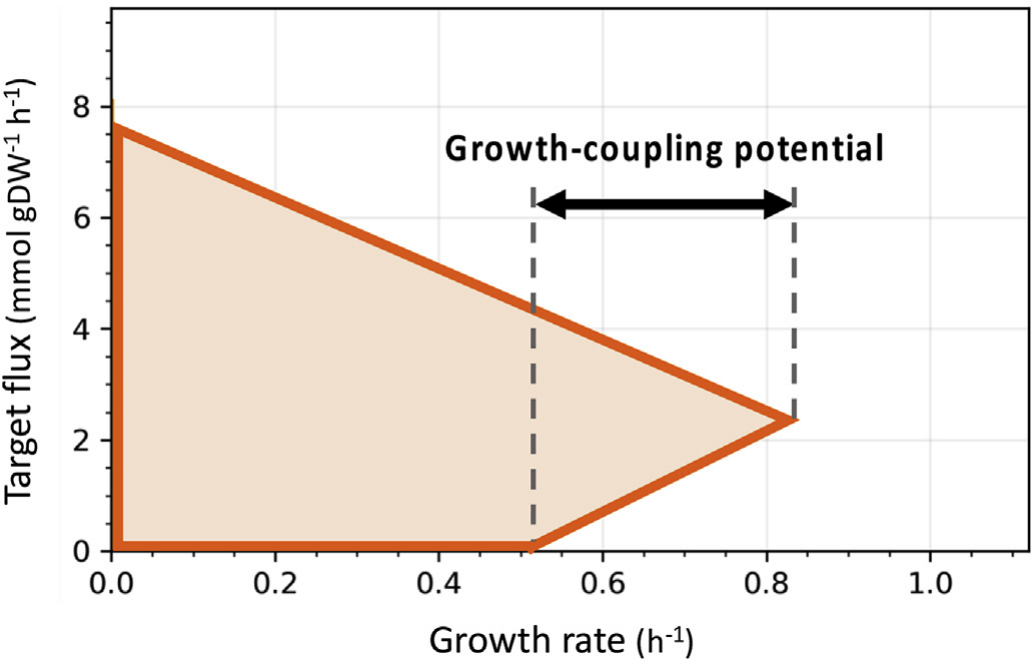
Visual depiction of the growth-coupling potential on a production envelope.

OptCouple is based on an MILP formulation, conceptually similar to the formulations used in existing algorithms like OptKnock, RobustKnock and SimOptStrain. MILP formulations are an efficient way of optimizing an objective function over a combinatorial space, such as the space of possible genetic modifications. The objective function of OptCouple is the growth-coupling potential (Fig. 1), defined as the amount with which the maximal growth rate will be decreased by preventing the target compound from being produced. Using the broadest definition of growth-coupling, sometimes called weak growth-coupling, namely that optimal growth requires a non-zero production flux (Feist et al., 2010; Klamt and Mahadevan, 2015), production is growth-coupled if and only if the growth-coupling potential is strictly positive. Optimizing for the growth-coupling potential ensures that the predicted strain designs and medium conditions will be easy to evolve with ALE to increase production, as the producing strains will have a large advantage over the non-producing strains. The algorithm RobustKnock maximises the minimum production at optimal growth instead, which also ensures growth-coupling, however the difference in growth rate between producers and non-producers can sometimes be marginal.

**Fig. 2:**
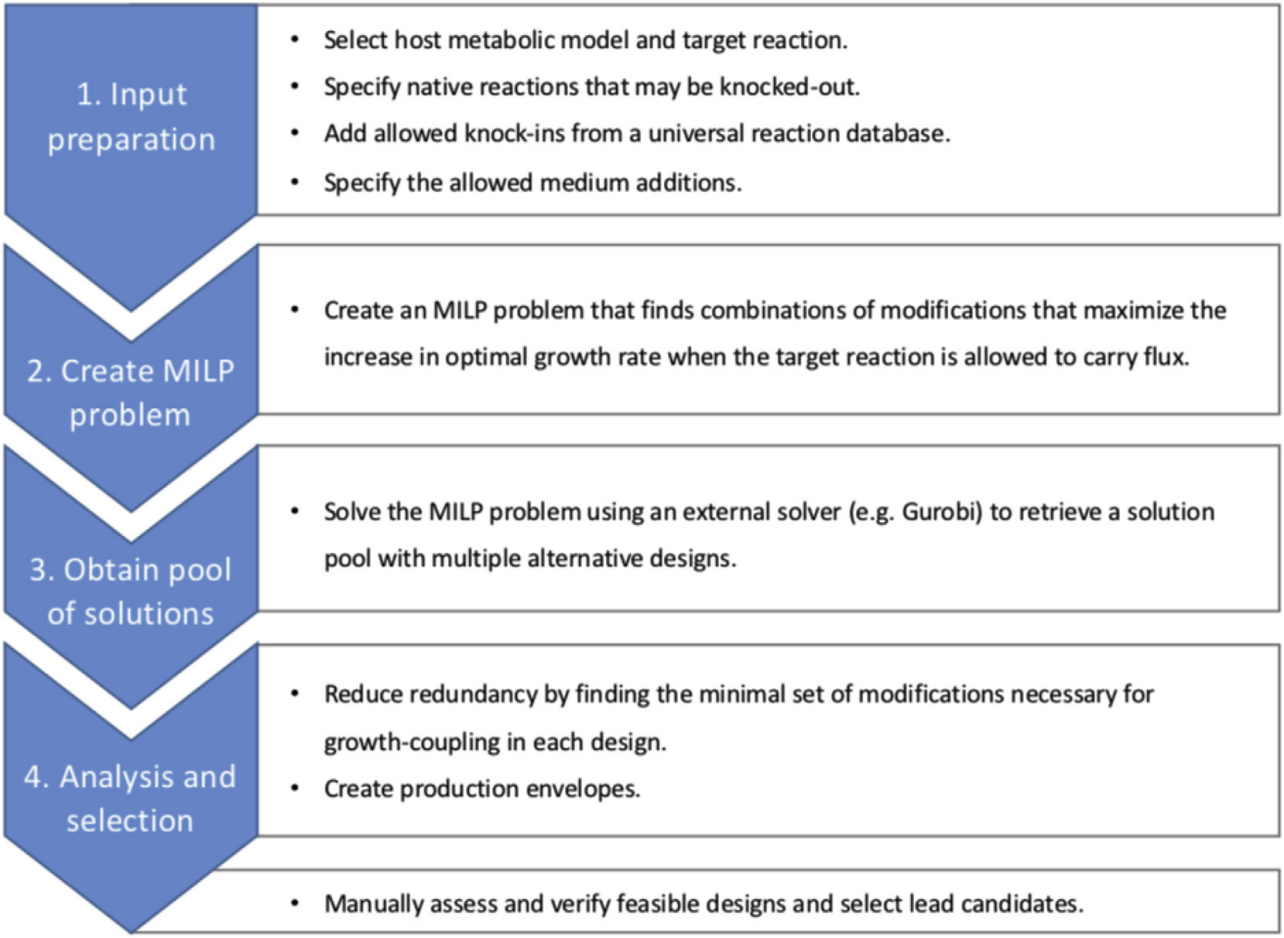
Overview of the workflow used for predicting growth-coupling designs with OptCouple.

Most previous methods try to find the single most optimal solution based on the chosen objective function. Since the most optimal solution (regardless of the objective function) might not be practically feasible for a strain engineering project, OptCouple uses an alternate approach to generate a large pool of different growth-coupled designs. These solutions can then be evaluated based on multiple parameters in order to find candidate strategies to implement *in vivo*. The workflow of OptCouple is shown in Fig. 2. In step 1, before running the MILP optimization, a metabolic model must be chosen, as well as the reaction to optimize. Furthermore, the universe of modifications must be defined. This includes deciding which native reactions may be knocked out, which heterologous reactions can be added, and which modifications to the medium are allowed. In step 2, the MILP problem is formulated, with binary variables to represent the allowed modifications. In step 3, the problem is solved using a dedicated MILP solver. Since the mathematically optimal solution is not necessarily the best strategy for a given metabolic engineering project, multiple solutions are identified in a single run, with high computational efficiency by using a solver with the capacity to find “solution pools”. Step 4 involves analysing the solutions found in step 3 and selecting one or more candidate strategies. Before manual inspection the number of solutions is automatically reduced by merging redundant solutions, i.e. separate solutions with only trivial differences, and ranking e.g. by growth-coupling potential or potential production rate.

## 4 Results and discussion

**Fig. 3:**
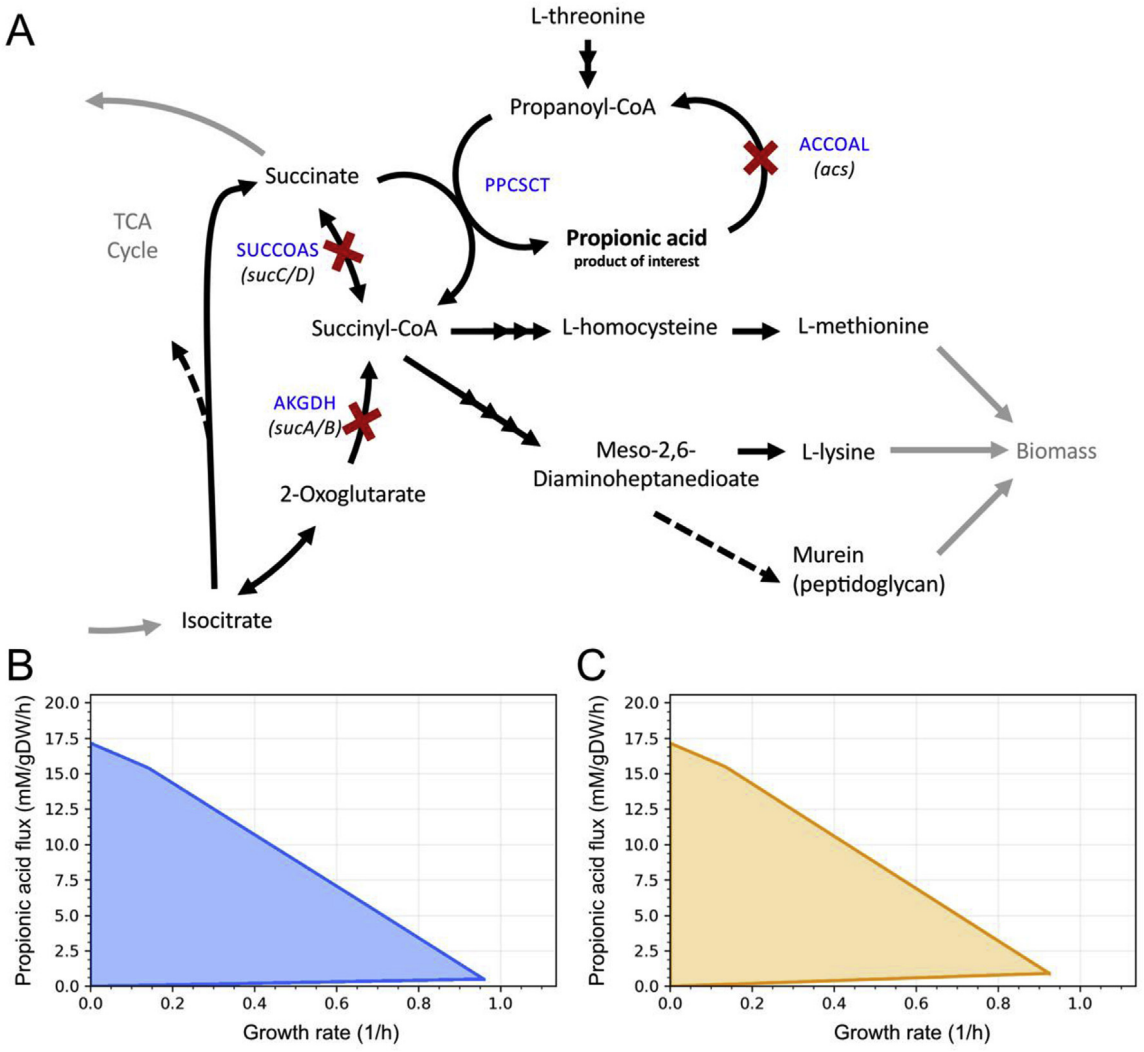
Overview of the designs predicted with OptCouple for growth-coupling of propionic acid. A) Pathway map of one of the predicted designs. Propionic acid production is coupled to succinyl-CoA production through the propanoyl-CoA succinate CoA-transferase. Alternative routes to succinyl-CoA are knocked out. B) Production envelope for the design shown in A. C) Production envelope of the second growth-coupled design, which couples production of propionic acid to the biosynthesis of NAD.

Initial testing of OptCouple was done to validate the novel objective function based on growth-coupling potential, and its ability to predict strain designs that are growth-coupled *in silico.* For this case, we chose propionic acid, which is an industrially relevant chemical that has not yet been produced biologically in economically viable amounts (Eş et al., 2017), and for which growth-coupling in *E. coli* has not been demonstrated. Furthermore, propionic acid is a native metabolite of *E. coli*, avoiding the necessity of first identifying or predicting a production pathway. OptCouple was run with a maximum of three knockouts, three insertions and one medium supplement, using a demand reaction for propionic acid as target. After removing redundancies in the predictions, two promising designs were identified, as seen in Table 1, which both produce propionic acid using the propionyl-CoA succinate CoA-transferase (PPCSCT) reaction. The first design, which is illustrated in Fig. 3, achieves growth-coupling by establishing propionic acid as a by-product of the supply of succinyl-CoA, which is a precursor for the biomass components methionine, lysine and murein. This is done by knocking out the native routes of producing succinyl-CoA (AKGDH and SUCOAS) as well as the recycling reaction for propionic acid (ACCOAL). The second design couples the PPCSCT reaction to the biosynthesis of NAD, establishing production of propionic acid as a by-product.

**Table 1:** Overview of selected predicted growth-coupling strategies for the three test cases. For each design is shown the required modifications, the production rate and yield at optimal growth (mmol/gDW/h and mol/mol glucose) and the growth-coupling potential, U, i.e. the difference in maximal growth rate between producers and non-producers. The knocked out and inserted reactions are denoted by their BIGG identifiers. The supplements are denoted by standard three-letter amino acid abbreviations.

Knockouts	Insertions	Supplements	Production rate	Yield	U
*Propionic acid:*					
ACCOAL, SUCOAS, AKGDH			0.50	0.05	0.95
MCITD, MTHFC, PFL			0.90	0.09	0.91

*Itaconic acid:*					
GLUDy, ICL, SUCOAS		L-glu	7.64	0.764	1.10
GLNS, ICL, SUCOAS		L-gln	0.24	0.024	1.11
ACKr, AKGDH, ICL, PGL, POX		L-ile	5.68	0.568	0.29
AKGDH, G6PDH2r, ICL, MDH, MGSA, PYK		L-asp	6.32	0.632	0.60

*Product methylation:*					
SERAT	CYSTL, CYSTGL	L-met	0.10	0.01	1.02
ASPTA	AHSERL2, HSERTA	L-met	2.69	0.269	0.97

Both of the strain designs for propionic acid lead to growth-coupling through non-obvious combinations of knockouts, but only require knockouts. To demonstrate the full potential of OptCouple and to test its ability to predict designs that are growth-coupled *in vivo*, we further evaluated the algorithm by its ability to identify known and experimentally validated growth-coupling strategies that require knockouts as well as medium supplements and gene insertions. We chose to use the itaconic acid growth-coupling of Harder et al. (2016) (requiring knockouts and medium supplement) as well as the product methylation growth-coupling of Luo and Hansen (2018) (requiring knockouts, medium supplement and gene insertions). Heterologous production of itaconic acid in *E. coli* can be achieved by the insertion of a single heterologous gene, *cadA* (*Aspergillus terreus*), encoding an enzyme that decarboxylates aconitic acid into itaconic acid (Harder et al., 2016). Growth-coupling has been realised by Harder et al. (2016) by knocking out the genes encoding isocitrate lyase, succinyl-CoA synthase, pyruvate kinase and phosphotransacetylase, as well as down-regulating isocitrate dehydrogenase. Additionally, Harder et al. (2016) inserted an orthologous citrate synthase to prevent allosteric regulation, but since the constraint-based modeling framework used here does not account for regulation, this modification was disregarded. When these modifications are applied to the iJO1366 genome-scale model of *E. coli* no growth-coupling is seen, as maximal growth does not allow for any production of itaconic acid. In order to attempt to reproduce the design, we chose to use the reduced metabolic model EColiCore2 (Hädicke and Klamt, 2017) instead. When the modifications from Harder et al. (2016) are introduced into this model, optimal growth does allow for production of itaconic acid, although it is not required.

**Fig. 4:**
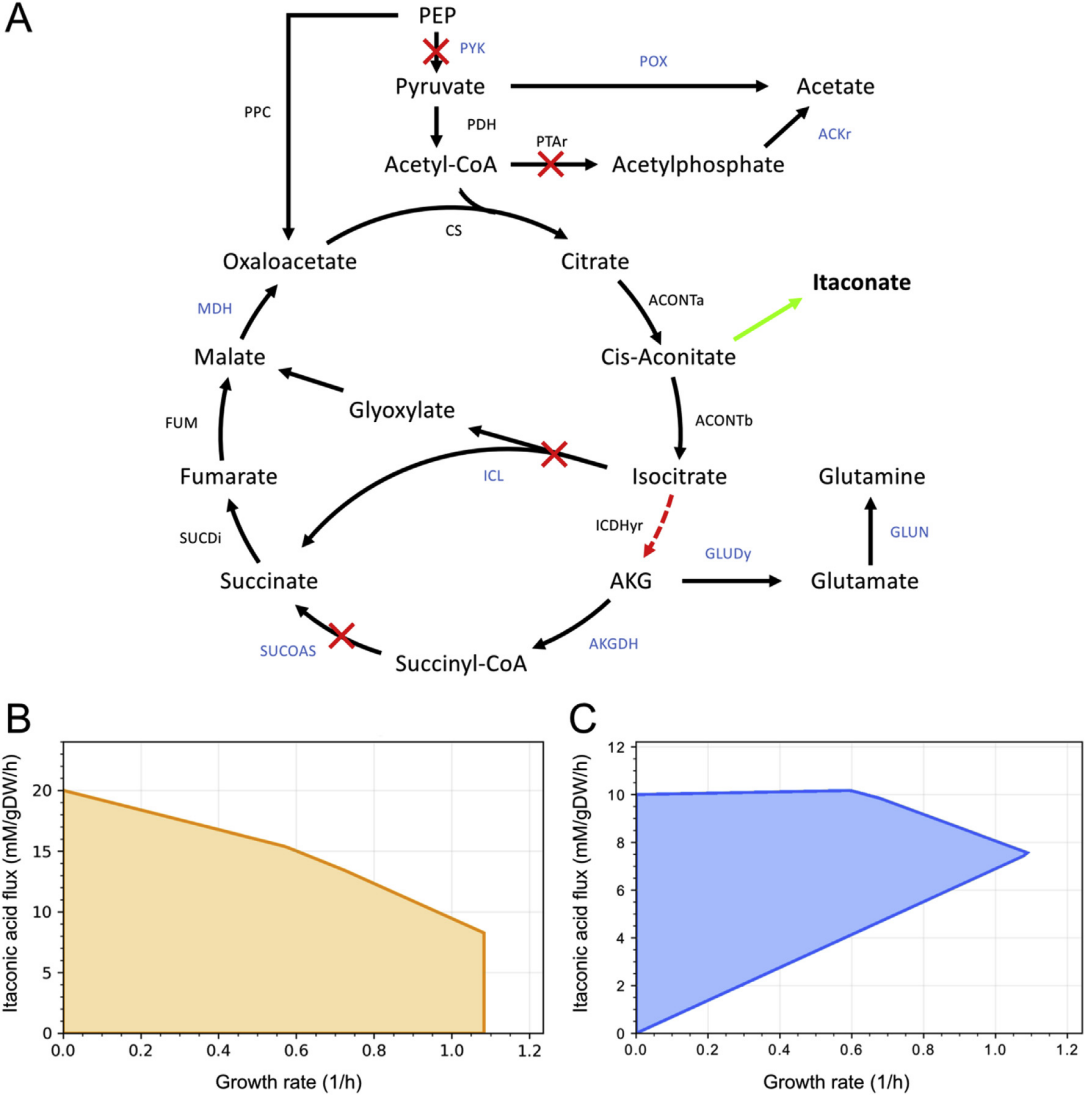
Overview of the itaconic acid growth-coupling designs. A) Metabolic map of reactions relevant to itaconic acid production. The red crosses are reactions that were knocked out by Harder et al. (2016). The reactions whose names are written in blue are reactions that were commonly knocked out in the designs predicted by OptCouple. B) Production envelope of the design by Harder et al. (2016). Knockouts: PYK, PTAr, ICL, SUCOAS. Medium supplement: L-glutamate. C) Production envelope of one of the growth-coupled designs predicted by OptCouple. Knockouts: ICL, SUCOAS, GLUDy. Medium supplement: L-glutamate. (For interpretation of the references to colour in this figure legend, the reader is referred to the Web version of this article.)

The itaconic acid-producing reaction was added to the model prior to running OptCouple, as the scope of this work was not to predict production pathways, but to identify growth-coupling strategies for an existing pathway. The algorithm was run, allowing up to six knockouts and a single medium supplement. A selection of the solutions is shown in Table 1. The majority of the identified designs contained modifications that are consistent with the design by Harder et al. (2016), as shown in Fig. 4. This includes disrupting the TCA cycle downstream of aconitate, the glyoxylate shunt, as well as reactions that can act as a sink for pyruvate or acetyl-CoA. Additionally, the algorithm suggested the addition of glutamate or glutamine to the medium, as also required in the design by Harder et al. (2016). The similarities between these results and the design by Harder et al. (2016) provided an indication that OptCouple can be used to predict combinations of knockouts and medium supplements and create functional strategies for coupling chemical production to growth.

While the results obtained for growth-coupling of itaconic acid demonstrated the utility of the algorithm for predicting knockouts and medium modifications, they did not require prediction of gene insertions. To test the ability of OptCouple to predict such modifications, the product methylation growth-coupling design of Luo and Hansen (2018) was used. This time the iJO1366 genome-scale model was chosen, as the modifications suggested by Luo and Hansen (2018) do confer growth-coupling in this context. To predict designs for product methylation, a dummy reaction converting SAM into *S*-adenosylhomocysteine (SAH) and an exportable methyl group metabolite was created and used as target reaction. The algorithm was run with a single knockout, two insertions and one medium supplement allowed. Among the predicted strategies we found a design that consisted of the exact same combination of modifications as suggested by Luo and Hansen (2018), while designs with several minor variations were also predicted. These variations consisted of different knockouts or insertions but resulted in the same general mechanism of growth-coupling, by requiring product methylation to convert SAM into SAH as part of the conversion of supplemented methionine into cysteine required for biomass production. The ability to predict the exact design of the validated methylation growth-coupling, as well as alternative seemingly equivalent designs, indicates that OptCouple can reliably be used to predict new feasible growth-coupling strategies, requiring a combination of gene knockouts, insertions and medium supplements.

**Fig. 5:**
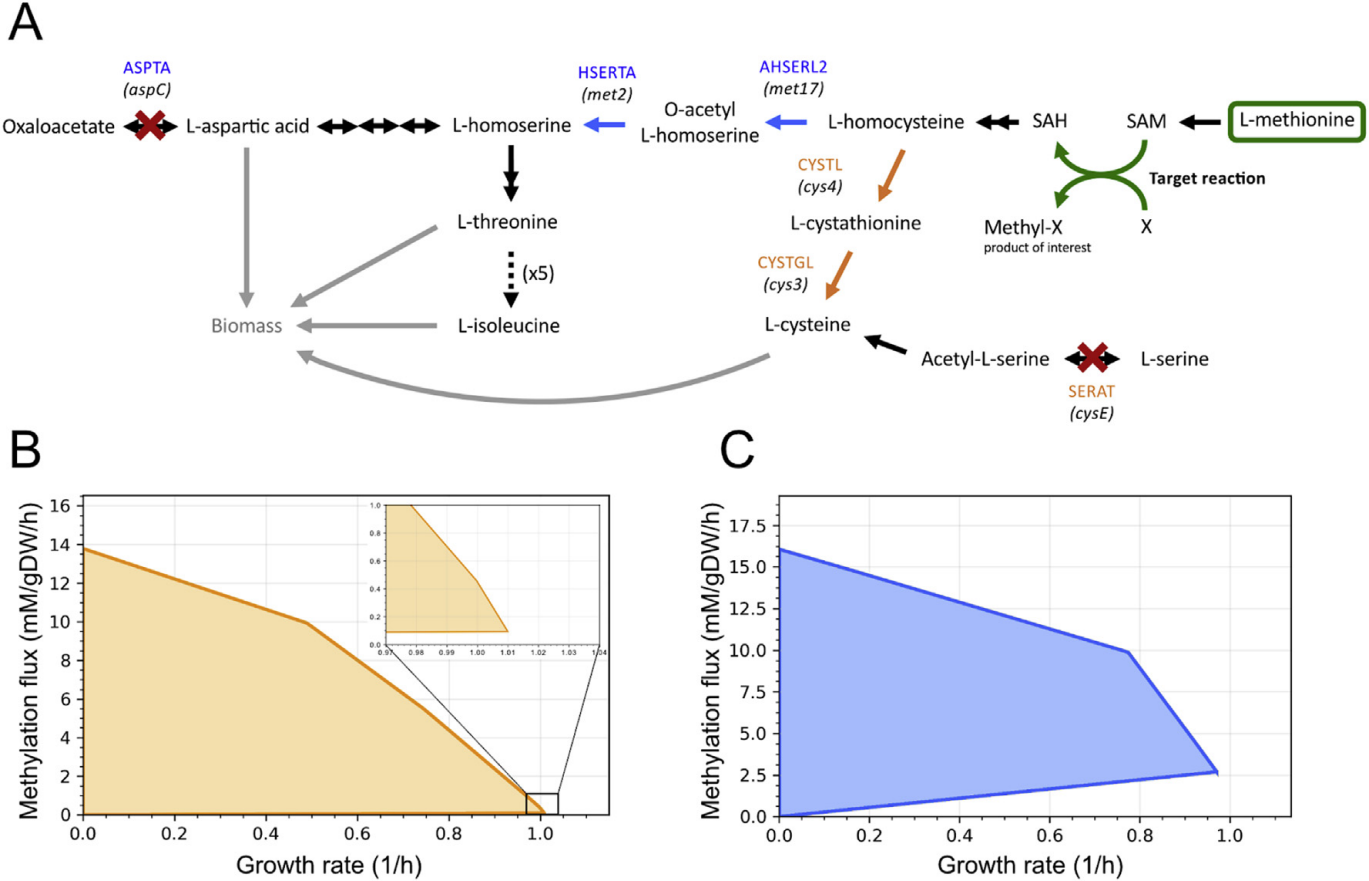
Overview of a subset of the predicted growth-coupling designs for product methylation. A) Pathway map showing the mechanisms of two growth-coupling strategies. The design of Luo and Hansen (2018) (orange) converts L-homocysteine into L-cysteine. The alternative design found here (blue) converts L-homocysteine in to L-threonine, L-isoleucine and L-aspartic acid. Both designs require supplementing the medium with methionine. B) Production envelope of the growth-coupling design of Luo and Hansen (2018). C) Production envelope of the alternative growth-coupling design found in this study. (For interpretation of the references to colour in this figure legend, the reader is referred to the Web version of this article.)

While the itaconic acid growth-coupling by Harder et al. (2016) results in a high production with yields of up to 0.68 mol/mol glucose, the methylation growth-coupling by Luo and Hansen (2018) has the disadvantage that only a relatively small flux is forced through the target pathway. Since methylation is required for the cell to synthesise cysteine, the growth-coupling will not drive methylation to exceed the cellular demand for cysteine which is quite low (Orth et al., 2011). We therefore used OptCouple to predict alternative growth-coupling strategies, which would be able to force a higher flux through the target methylation reaction. One such strategy was discovered, that uses product methylation to convert supplemented methionine into the amino acids aspartate, threonine and isoleucine, while disabling the native production of these. This will demand a higher flux through the methylation reaction at a given growth rate than the original design coupling methylation to cysteine biosynthesis. Fig. 5 shows the two growth-coupling designs and their respective production envelopes. The production envelope for the alternative design (Fig. 5C) shows a larger potential production rate by growth-coupling (indicated by the height of the right-most point) than the original design (Fig. 5B), consistent with the combined higher cellular demand for aspartate, threonine and isoleucine compared to cysteine (Orth et al., 2011).

The above results prove that OptCouple can be used to identify combinations of knockouts, gene insertions and medium supplements that make production of various compounds coupled to growth in *E. coli*. The algorithm could easily find designs allowing up to 7 modifications with running times less than 24 h. The fact that OptCouple identifies designs that are identical or very similar to prominent, experimentally validated growth-coupling designs indicates that it will also be able to find novel valid growth-coupling designs.

The main novelty and advantage of OptCouple is the possibility of simultaneously identifying complex combinations of three different types of modifications. Currently, other strain design algorithms exist that attempt to find growth-coupled designs through the identification of one or two types of modifications simultaneously. Recent examples are SimOptStrain (Kim et al., 2011) that simultaneously identifies gene knockouts and insertions, whereas SelFi (Hassanpour et al., 2017) can suggest all three types of modifications, but only medium supplements and gene knockouts are identified simultaneously. Several successful designs, however, such as the product methylation growth-coupling (Luo and Hansen, 2018), show that considering all three types of modifications at once can enable the identification of new growth-coupling strategies.

OptCouple guarantees that the resulting designs are truly growth-coupled. This is in contrast to e.g. SimOptStrain, which uses the same objective function as OptKnock, and thus does not specifically predict growth-coupling, as competing pathways are still allowed. Ideally, a growth-coupled strain design should have a high growth-coupling potential as well as a high production rate. Thus, a potential drawback of using the growth-coupling potential as objective function in OptCouple is that there is no explicit optimization of the production rate that can be achieved by growth-coupling. This can result in predictions with a robust growth-coupling, but only very small production flux. An example of this issue is seen in the identified growth-coupling strategies for propionic acid. The design identified by OptCouple ensures the production of propionic acid to supply the cell with either NAD or methionine, lysine and murein, all of which are only needed in relatively small amounts. The consequence is that the growth-coupled production rate of propionic acid will not be sufficient for a commercially viable process, given the modest market price of propionic acid (Rodriguez et al., 2014). Even though this limits the practical utility of some growth-coupling strategies identified by OptCouple, it does not significantly reduce the utility of the algorithm itself. While a high growth-coupling potential is no guarantee of a high production rate, it also does not prohibit it. Through the use of suboptimal solution pools, OptCouple can quickly identify many candidate designs, increasing the likelihood that at least one will have robust growth-coupling and have a high growth-coupled production rate. Additionally, computationally predicted strain designs should always be assessed manually before being implemented in the laboratory, as their feasibility can also be affected by a range of factors not considered in the models, e.g. thermodynamics, regulation, toxicity, etc, which further underlines the value of evaluating a large set of alternative designs.

As with all model-based predictions, the quality of the results strongly depends on the quality of the model that was used. As one of the most commonly used organisms for metabolic modeling, the *E. coli* genome-scale model is relatively comprehensive. While nothing prevents OptCouple from being used in other organisms, the predicted designs should be curated even more thoroughly if a less complete metabolic model is used. The *in vivo* presence of enzymes that are not accounted for in the model can effectively abolish the growth-coupling of a predicted design, as they can allow the cell to circumvent the growth-coupling mechanism. Conversely, if a model contains reactions that are not active *in vivo*, e.g. due to transcriptional repression, some growth-coupling strategies will require more modifications *in silico* than they would in practice. This is seen in the experimentally validated itaconic acid growth-coupling design (Harder et al., 2016), which does not show growth-coupling when simulated with iJO1366, whereas the reduced model EColiCore2 did allow production at optimal growth. However, during optimization with ALE, repressed reactions could become active allowing the cell to circumvent growth-coupling mechanisms predicted with reduced models. Therefore, it would most likely be preferable to use the most complete model available for the chosen organism.

## 5 Conclusion

OptCouple is an MILP-based optimization algorithm that can find combinations of gene knockouts, heterologous gene insertions, and additions to the growth medium, that allow the stoichiometric coupling of a product of interest to growth. In our validation tests OptCouple was able to reproduce successful growth-coupling designs from the published literature and find alternative designs that allow for a higher production flux. Furthermore, we showed that OptCouple can be used to predict novel candidate growth-coupling designs for target compounds where no growth-coupling has previously been demonstrated.
