# Supplementary material for: OptCouple: Joint simulation of gene knockouts, insertions and medium modifications for prediction of growth-coupled strain designs
Source: Metab Eng Commun. 2019 Mar 16;8:e00087. doi: 10.1016/j.mec.2019.e00087 (PMC6431744; doi:10.1016/j.mec.2019.e00087)
Supplement: Multimedia component 1 [file mmc1.zip › Revised Supplementary/Mathematical formulation of OptCouple.docx]

**Mathematical formulation of OptCouple**

The metabolic model used in OptCouple is given by a set of metabolites $m_{i}\forall i\in N$, and a set of metabolic reactions $r_{j} \forall j\in R$. A stoichiometric matrix *S* encodes which metabolites participate in each reaction (Orth et al., 2010). R is partitioned by the three subsets, $R_{native}$, $R_{heterologous}$ and $R_{additions}$, representing native reactions, heterologous reactions and boundary reactions for potential medium additions, respectively. Furthermore, some reactions $r_{j} \forall j\in R_{irreversible}$ can only proceed in the forward direction, while the remaining reactions can proceed in both directions. Each reaction is associated with a binary control variable, $y_{j}\in Y \forall j\in R$.

The primal problem (M) optimizes biomass production subject to stoichiometric constraints, limited glucose uptake and genetic modifications, Y:

$Maximise_{\boldsymbol{v}} v_{biomass}$ (S1)

subject to:

$\sum_{j\in R} s_{ij}\cdot v_{j}=0 \forall i\in N$

$v_{j}^{min}\cdot y_{j}\leq v_{j}\leq v_{j}^{max}\cdot y_{j} \forall j\in R$

$v_{glc\_uptake}\leq10$

$v_{j}\geq0 \forall j\in R_{irreversible}$

$y_{j}\in\left\{ 0, 1 \right\}, \forall j\in R$

$\sum_{j\in R_{native}} (1-y_{j})\leq K_{native}$

$\sum_{j\in R_{heterologous}} y_{j}\leq K_{heterologous}$

$\sum_{j\in R_{additions}} y_{j}\leq K_{additions}$

The problem can be modified to not allow flux in the target reaction $r_{target}$, resulting in M*:

$Maximise_{\boldsymbol{v}} v_{biomass}$ (S2)

subject to:

$\sum_{j=1}^{|R|} s_{ij}\cdot v_{j}=0, \forall i\in N$

$v_{j}^{min}\cdot y_{j}\leq v_{j}\leq v_{j}^{max}\cdot y_{j}, \forall j\in R$

$v_{target}=0$

$v_{glc\_uptake}\leq10$

$v_{j}\geq0, \forall j\in R_{irreversible}$

$y_{j}\in\left\{ 0, 1 \right\}, \forall j\in R$

$\sum_{j\in R_{native}} (1-y_{j})\leq K_{native}$

$\sum_{j\in R_{heterologous}} y_{j}\leq K_{heterologous}$

$\sum_{j\in R_{additions}} y_{j}\leq K_{additions}$

M* can then be converted to its dual form, $M_{D}^{*}$ (as described by Burgard et al. (2003)):

$Minimise_{\boldsymbol{\mu}\boldsymbol{,}\boldsymbol{\lambda}} 10\cdot\mu_{glucose\_uptake}$ (S3)

subject to:

$\sum_{i=1}^{\left| N \right|} \lambda_{i}^{stoich}\cdot s_{ij}+\mu_{j}=0, \forall j\in R, j\neq biomass$

$\sum_{i=1}^{|N|} \lambda_{i}^{stoich}\cdot s_{i,biomass}+\mu_{biomass}=1$

$\mu_{j}^{min}\cdot\left( 1-y_{j} \right)\leq\mu_{j}\leq\mu_{j}^{max}\cdot\left( 1-y_{i} \right), \forall j\in R, j\neq target$

$y_{j}\in\left\{ 0, 1 \right\}, \forall j\in R$

$\sum_{j\in R_{native}} (1-y_{j})\leq K_{native}$

$\sum_{j\in R_{heterologous}} y_{j}\leq K_{heterologous}$

$\sum_{j\in R_{additions}} y_{j}\leq K_{additions}$

Here $\lambda_{i}^{stoich}$ represent dual variables of the stoichiometric constraints in the primal, while $\mu_{i}$ represent other flux bounds. The minimum and maximum values, $\mu_{j}^{min}$ and $\mu_{j}^{max}$ as well as $v_{j}^{min}$and $v_{j}^{max}$can be found by sequentially minimizing and maximizing the variables or by using a sufficiently large constant (the big-M method).

The two problems $M$ and $M_{d}^{*}$ are combined and optimized simultaneously, together with the binary variables Y:

$Maximise_{\boldsymbol{v}\boldsymbol{,}\boldsymbol{\lambda}\boldsymbol{,}\boldsymbol{\mu}\boldsymbol{,}\boldsymbol{Y}} v_{biomass}-10\cdot\mu_{glucose\_uptake}$ **OptCouple** (S4)

subject to:

$\sum_{j=1}^{|R|} s_{ij}\cdot v_{j}=0 \forall i\in N$

$v_{j}^{min}\cdot y_{j}\leq v_{j}\leq v_{j}^{max}\cdot y_{j} \forall j\in R$

$v_{glc\_uptake}\leq10$

$v_{j}\geq0 \forall j\in R_{irreversible}$

$\sum_{i=1}^{\left| N \right|} \lambda_{i}^{stoich}\cdot s_{ij}+\mu_{j}=0, \forall j\in R, j\neq biomass$

$\sum_{i=1}^{|N|} \lambda_{i}^{stoich}\cdot s_{i,biomass}+\mu_{biomass}=1$

$\mu_{j}^{min}\cdot\left( 1-y_{j} \right)\leq\mu_{j}\leq\mu_{j}^{max}\cdot\left( 1-y_{i} \right), \forall j\in R, j\neq target$

$y_{j}\in\left\{ 0, 1 \right\}, \forall j\in R$

$\sum_{j\in R_{native}} (1-y_{j})\leq K_{native}$

$\sum_{j\in R_{heterologous}} y_{j}\leq K_{heterologous}$

$\sum_{j\in R_{additions}} y_{j}\leq K_{additions}$

Optimizing (S1) finds the highest possible growth rate of the organism and the modifications necessary to achieve this. Similarly, optimizing (S2) or (S3) finds the highest growth rate possible with no flux through the target reaction. Jointly solving (S1) and (S3) with shared binary variables, as in (S4), finds the highest difference between maximal growth rates with and without flux through the target reaction (and the required combination of binary variable values). For a target reaction representing production, this difference corresponds to the growth-coupling potential, i.e. the maximal growth advantage of producer cells compared to non-producer cells. Any combination of binary variable values that results in a non-zero growth-coupling potential corresponds to a (weakly) growth-coupled strain design.

A design with high growth-coupling potential will be easier to evolve using ALE, compared to designs with lower growth-coupling potentials, due to the larger potential increase in growth rate. However, since a high growth-coupling potential does not guarantee a high growth-coupled production rate, designs with sub-optimal growth-coupling potentials might be preferable. Such sub-optimal solutions can be sampled using the solution pool feature of some commercial MILP solvers (e.g. Gurobi or CPLEX).

**References:**

Burgard, A.P., Pharkya, P., Maranas, C.D., 2003. OptKnock: A Bilevel Programming Framework for Identifying Gene Knockout Strategies for Microbial Strain Optimization. Biotechnol. Bioeng. 84, 647–657. https://doi.org/10.1002/bit.10803

Orth, J.D., Thiele, I., Palsson, B.Ø., 2010. What is flux balance analysis? Nat. Biotechnol. 28, 245–248. https://doi.org/10.1038/nbt.1614
